# A comparison of Lyse-It to other cellular sample preparation, bacterial lysing, and DNA fragmentation technologies

**DOI:** 10.1371/journal.pone.0220102

**Published:** 2019-07-23

**Authors:** Tonya M. Santaus, Shan Li, Lahari Saha, Wilbur H. Chen, Siya Bhagat, O. Colin Stine, Chris D. Geddes

**Affiliations:** 1 Chemistry and Biochemistry Department, University of Maryland, Baltimore County, Baltimore, MD, United States of America; 2 Institute of Fluorescence, University of Maryland, Baltimore County, Baltimore, MD, United States of America; 3 Epidemiology and Public Health Department, University of Maryland School of Medicine, Baltimore, MD, United States of America; 4 Center for Vaccine Development and Global Health, University of Maryland School of Medicine, Baltimore, MD, United States of America; Defense Threat Reduction Agency, UNITED STATES

## Abstract

The ability for safe and rapid pathogenic sample transportation and subsequent detection is an increasing challenge throughout the world. Herein, we describe and use bead-beating, vortex, sonication, 903 protein saver cards, and Lyse-It methods, aiming to inactivate Gram-positive and -negative bacteria with subsequent genome DNA (quantitative Polymerase Chain Reaction) qPCR detection. The basic concepts behind the four chosen technologies is their versatility, cost, and ease of use in developed and underdeveloped countries. The four methods target the testing of bacterial resilience, cellular extraction from general and complex media and subsequent DNA extraction for qPCR detection and amplification. These results demonstrate that conventional high temperature heating, 903 protein saver cards, and Lyse-It are all viable options for inactivating bacterial growth for safe shipping. Additionally, Lyse-It was found to be particularly useful as this technology can inactivate bacteria, extract cells from 903 protein saver cards, lyse bacterial cells, and additionally keep genomic DNA viable for qPCR detection.

## Introduction

One of the hindrances to rapid detection of pathogenic bacteria and allocation of preventative and diagnostic care to patients around the world is the lack of readily available medical centers, trained physicians, and diagnostic platforms.[1, 2] For patients in rural areas, especially in third world countries, the ability to get to a medical center is difficult. Patients or physicians must travel long distances or establish alternative means for the diagnosis and treatment of a wide variety of diseases.[3] Telemedicine and telepathology are two of the ways to aid in solving this problem, as these are methods where visual information and data can be transmitted digitally, through secure web-based systems like what’s app, instant messenger systems, email, and safely through the mail. Biological materials and samples are broken down into various categories of risk, like Category A, which is biological material that is highly likely to cause fatalities or significant harm to humans. Category B biologicals pertains to samples that have the potential to cause moderate harm to humans and animals. Currently, pathological specimens containing live or dangerous substances to humans or animals require special handling, packaging, and tightly regulated shipping procedures. Individual shipping carriers like USPS[4], UPS[5], FedEx[6], and many airlines[7], have their own rules and regulations on what can and cannot be shipped, how the materials need to be packaged, and what documentation is required. For example, according to FedEx for Category B biological samples (Code UN 3373), samples need to be contained in a watertight leak-proof primary receptacle where the sample cannot exceed 1 L in total volume. In between the primary and secondary container much be a cushioning liquid absorbent material and the primary and/or secondary container must be able to withstand pressure and temperature differentials. The outer packaging must be ridged consisting of specified materials and be appropriately sized for the internal contents. Finally, the package needs to be labeled according to IATA and OSHA guidelines[7, 8] with the correct information inside the package and given to the shipping and handling company.[6] Two ways to reduce the need for special shipping procedures of pathogenic and biological samples is through the inactivation of pathogens using RNAlater or an equivalent reagent, or with the use of a dry sample transport and storage platform. One dry sample platform that fits these requirements are the 903 protein saver cards. 903 protein saver cards have been routinely used to date for malaria blood spots and more recently for stool samples by the Pasteur Institute, The Sanger Center, Johns Hopkins, and the US Army. Since the gold standard for pathogen detection is through culture, these protein saver cards have been used for storage of various different samples.[9–12] These cards have the advantage of being able to have biological specimens transported at room temperature without being treated as a biohazard, can be archived for 17 years until processing (real-time stability with human blood on FTA), and only requires an FTA purification reagent or water for cell/DNA elution.[13]

Herein we describe and compare four methods–bead-beating, vortex, sonication, and Lyse-It—for the extraction of pathogenic bacteria from raw samples and from 903 protein saver cards for their subsequent detection through quantitative Polymerase Chain Reaction (qPCR). The Lyse-It technology has been extensively reviewed and used for a variety of applications including pathogenic bacteria and STI/STD cellular lysing.[14–18] Additionally, we assess these methods for safe handling and shipping without the need for potential regulations because biomolecules like DNA and dead bacteria are not pathogenic and do not pose a threat to humans or animals. Methods like bead-beating[19, 20], Lyse-It, and 903 protein saver cards[13] allow for the safe preparation of hazardous biological materials to be shipped safely for analysis. In particular, Lyse-It and 903 protein saver cards can be used anywhere, require only stable reagents, produce samples that are ready for diagnostic analysis, and *most importantly*, are safe to ship. Additionally, samples can be shipped on 903 protein saver cards through various methods of transportation. The other methods like vortex, sonication, Lyse-It, and bead-beating can be used for the extraction of the sample from the card for future detection methods like (Nucleic Acid Amplification Tests) NAAT, sequencing, (Enzyme-Linked Immunosorbent Assays) ELISAs, and (quantitative Polymerase Chain Reaction) qPCR. Lyse-It has the additional advantage of being able to render the growth of bacteria inactive and thus safe to ship as well as enabling the extraction, cellular lysis, and subsequent DNA fragmentation for downstream detection methods like qPCR from 903 protein saver cards.

## Materials and methods

*Vibrio cholerae* patient fecal samples were partitioned out onto Watman 903 protein saver cards and into microcentrifuge tubes using the stool weights shown in **Table 1**. Subsequently, varying concentrations (10^0^–10^8^ CFU/mL) of *Listeria monocytogenes* or *V*. *cholerae* suspended in DI water and a 60 μL aliquot of the suspension was added to each circle of the 903 protein saver Cards. The samples on the protein saver cards are known as filter paper (FP) and the samples in the microcentrifuge tubes are described as raw samples for the experiments. A visual breakdown of the following procedures; bead-beating, vortex, protein saver cards, and Lyse-It is shown in **S1 Schematic**.

**Table 1:** Amount needed for sample processing of various *V*. *cholerae* stool grades.

Stool Grade	*V*. *cholerae* Plate Count (CFU/gram)	Amount Needed for Plate Count	Raw Sample Amount	Filter Paper Amount
1	0	1 gram	30 mg ± 1.4 mg	50 mg
3	10^6^	1 mL or 1 gram	28 mg ± 4.2 mg	50–60 mg
4	10^7^	1 mL	22.8–36 mg	40–50 mg
5	10^7^−10^9^	1 mL	23–44 mg	30–40 mg

### *V*. *cholerae* plate counting

Solid to semi-solid (1 gram) stool was weighed out and suspended into 9 mL of normal saline. Dilutions (10^−2^, 10^−4^, and 10^−5^) were plated on Thiosulfate, Citrate, Bile Salts, Sucrose (TCBS) plates (Fisher Scientific R01865) using a spreader. If stool specimens were liquid, 1 mL of the stool was mixed with 9 mL of normal saline and dilutions of 10^−4^, 10^−5^, and 10^−6^ were plated on TCBS plates. For the dilutions, the first dilution was performed in a 50 mL falcon tube and used for subsequent dilutions in a 48-well microtiter plates with 100 μL of specimen to 900 μL of saline. Mixing was performed vigorously by pipetting up and down at least 30 times. Growth colonies were counted after incubation. To confirm that the colonies were *V*. *cholerae*, suspected colonies were transferred onto LB or TSA agar plates (Fisher Scientific) for performing the oxidase test and serological testing.

### 903 protein saver card standard processing

**Fig 1:**
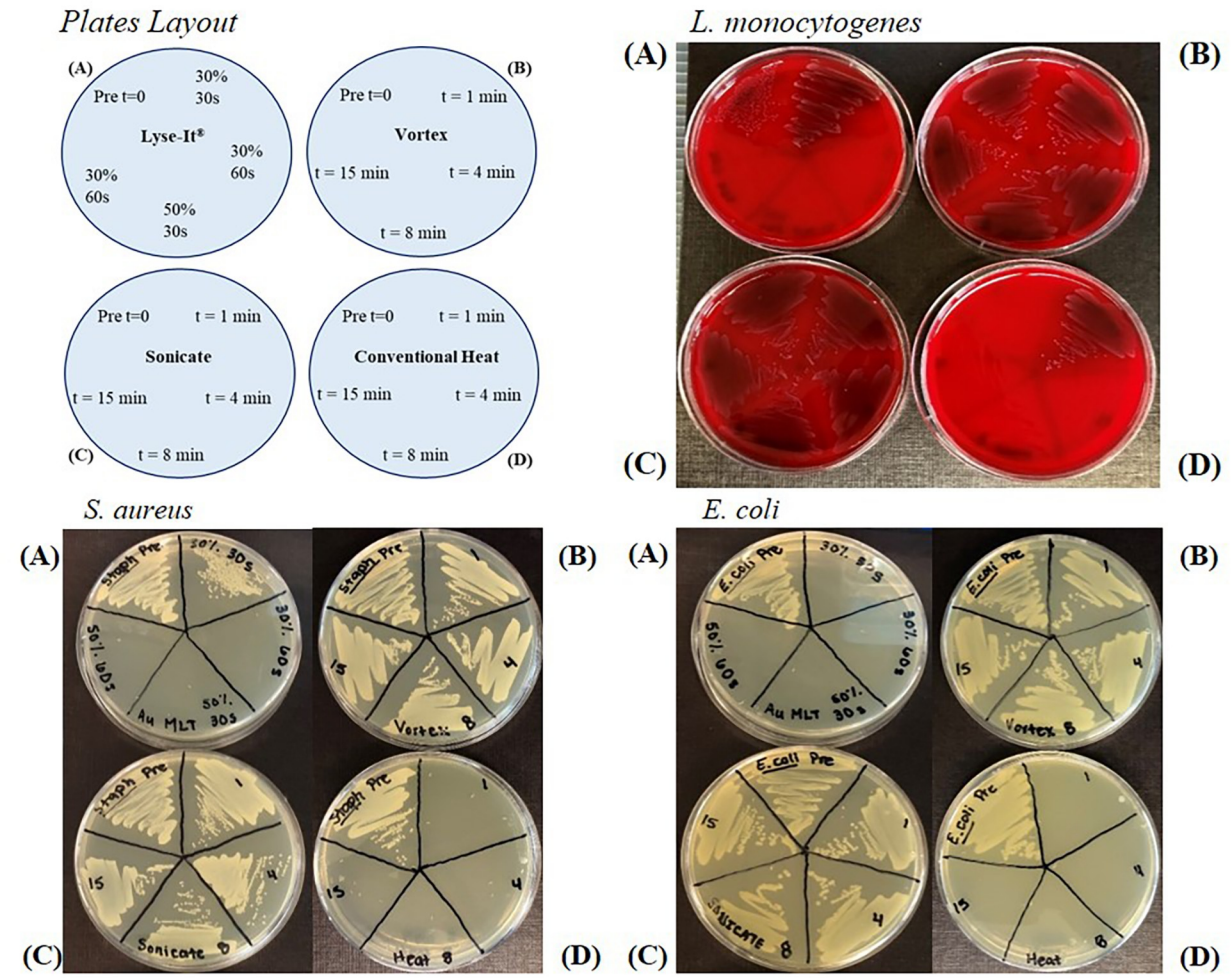
General layout of bacterial growth plates (Top Left). *L*. *monocytogenes* (Top Right) *S*. *aureus* (Bottom Left) and *E*. *coli* (Top Right) growth following various cellular disruption techniques including Lyse-It **(A)**, vortex **(B)**, sonication **(C)**, and 60ºC conventional heating **(D)**. *L*. *monocytogenes* growth is inactivated following microwave irradiation of 30% power for 60 seconds or with conventional heating for over 1 minute. *S*. *aureus* growth is inactivated following microwave irradiation of 30% power for 60 seconds or with conventional heating for over 1 minute. *E*. *coli* is inactivated following 30% power for 30 s seconds and over 4 minutes of conventional heating. In all cases, 15 minutes of vortex or sonication does not result in any significant growth inhibition for *L*. *monocytogenes*, *V*. *cholerae*, *S*. *aureus*, or *E*. *coli*. Pre = 10^8^ CFU/mL suspension without further processing.

One circle from the protein saver card or the amount of stool grade reported in **Table 1** was cut out and placed into a 1.5 mL labeled microcentrifuge tube. The varying color of the different stool grades as raw samples and on 903 protein saver cards can be seen in **S1 Fig**. 1 mL of 1x Phosphate Buffer Saline (PBS) was added to the tube and incubated at room temperature for 10 minutes. Post incubation, the samples were centrifuged for 2 minutes at 14,000 rpm. The supernatant was discarded and 200 μL of DI water was added to the pellet and a saver card circle. The samples were incubated in a 100°C heating block for 8 minutes. Upon cooling to room temperature, the samples were centrifuged through Costar Spin-X 0.45 μm cellulose acetate RNase/DNase free centrifuge tube filters for 1–3 minutes at 14,000 rpm. The centrifugation time can be adjusted so that all the sample volume can be filtered through the cellulose filter. The extracted samples were stored at -20°C until qPCR analysis.

### Bead-beating process

Screwcap 2.0 mL bead-beater sample tubes (one for each sample) were prepared with 1 mL 0.05 M phosphate buffer and 4 glass beads (3mm). 200–300 mg solid stool of up to 500 μL loose stool was placed into the tubes and kept on ice. Each sample was vortexed for 1–2 minutes or until thoroughly homogenized. Post homogenization, samples were centrifuged for 2 minutes at full speed (16,000 g). The supernatant was discarded and 1.4 mL ASL buffer from the QIAamp kit was added to each tube and vortexed continuously for 1 minute. Following vortex, samples were heated in a water bath at 70°C for 5 minutes. Approximately 0.3 grams of 0.1 mm zirconium beads were added, and the sample was “beat” for 3 minutes using a bead-beater speed for effective homogenization. To pellet the particles, samples were centrifuged for 1 minute at full speed. Without disturbing the pellet, 1.2 mL of supernatant was collected into a new microcentrifuge tube and the pellet was discarded. To the extracted supernatant, one InhibitEX tablet was added and vortexed immediately for 1 minute or until the tablet was completely suspended. To allow for complete absorption of the tablet, samples were incubated at room temperature for 3 minutes. After incubation, samples were centrifuged at full speed for 3 minutes. All supernatant was collected into a new centrifuge tube and was considered ***complete lysate***. The pellet was discarded. In a new 1.5 mL microcentrifuge tube, 25 μL Proteinase K (QIAamp kit), 400 μL AL buffer (QIAamp kit), and 400 μL of *complete lysate* was added. This solution was vortexed for 15 seconds and incubated in a 70°C water bath for 10 minutes. 400 μL of 100% ethanol was added to the lysate and vortexed for 15 seconds (ethanol lysate). A QIAamp spin column was labeled and 610 μL of the ethanol lysate was added and centrifuge at full speed for 1 minute. The collection tube was discarded. The spin column was added to a new collection tube and the remaining ethanol lysate was centrifuged. The collection tube was discarded, and the sample was added to a new collection tube. To the spin column 500 μL QIAamp kit AW2 buffer was added and centrifuge at full speed for 3.5 minutes. The filtrate was discarded, and the spin column was placed into a new tube. QIAamp kit AE buffer was preheated at 65°C for 5–10 minutes. Once AE buffer was warmed, 200 μL AE buffer was added to the spin column membrane, incubated at room temperature for 10 minutes, and then centrifuge at full speed for 3 minutes to elute DNA. The filtrate was reloaded and centrifuged again for 3 minutes. DNA concentration was measured on a Nanodrop instrument and if the DNA quality was poor, then ethanol precipitation was performed using the extracted DNA.

### Ethanol precipitation of extracted DNA following bead-beating

In a 1.5 mL microcentrifuge tube, 1/10 volume of 3M sodium acetate (sterile pH 5.2) and 200 μL of DNA solution was added and briefly vortexed. Approximately 500 μL of 100% ice cold ethanol was then added and mixed by inverting 5 times. Mixed solutions were stored in a -70°C freezer for 15 minutes or 30 minutes in a -20°C freezer. Cold samples were centrifuged at full speed (16,000 rpm) for 15 minutes to pellet the DNA. The supernatant was removed and 500 μL of 70% ethanol was added. The samples were vortexed for 1 minute followed by centrifugation at full speed for 15 minutes. The supernatant was removed, and the pellet dried at room temperature. Following drying, 50–100 μL 1x TE buffer was added to the dried pellet, vortexed, briefly spun down, to allow the DNA to dissolve thoroughly before the OD was measured.

### Vortex processing

One circle from the protein saver cards or the amount of stool grade reported in **Table 1** was cut out and/or placed into a 1.5 mL labeled microcentrifuge tube. 1.25 mL of DI water was added to rehydrate the saver card circle and subsequent cells and incubated at room temperature for 10 minutes. Samples were centrifuged for 2 minutes at 14,000 rpm. The supernatant was discarded, and 1 mL of DI water was added to the pellet. Samples were then vortexed for 5 minutes. The mixed samples were extracted out and placed into a Costar Spin-X 0.45 μm cellulose acetate centrifuge filter. Samples were centrifuged at 14,000 rpm for 1 minute or until all liquid had been removed from the filter. The filter paper was discarded, and the sample filtrate was stored at -20°C until qPCR analysis.

### Lyse-It processing

One circle from the protein saver cards or the amount of stool grade reported in **Table 1** was cut out and/or placed into a 1.5 mL labeled microcentrifuge tube. 1.25 mL of DI water was added, and the samples were incubated at room temperature for 10 minutes. Following incubation, samples were centrifuged for 2 minutes at 14,000 rpm and the supernatant then discarded. 1.25 mL of DI water was added to the pellet/ protein card circle and mixed/ suspended through pipetting until solution became dirty/ opaque. 1mL of suspended sample was aliquoted onto a Lyse-It slide enclosed sample chamber and a slide cover was adhered to prevent sample loss and contamination. (See www.Lyse-It.com) Samples were microwave irradiated for 60 seconds at 30% power (270 W) in a Frigidaire 900W microwave equipped with a slide holder. After microwave irradiation, sample was taken up and placed into a Costar Spin-X 0.45 μm cellulose acetate centrifuge filter and centrifuged for 1–3 minutes or until all liquid had been removed from the top of the filter. The same centrifuge filter can be used, and the sample filtrates can be combined into 1 microcentrifuge tube for storage. All samples were stored at -20°C until qPCR analysis.

### Quantitative polymerase chain reaction (qPCR)

Quantitative polymerase chain reaction (qPCR) was performed in the Department of Epidemiology and Public Health at the University of Maryland School of Medicine. Samples were prepared as described above. An ABI QuantStudio 3 qPCR was used with the following primers for *L*. *monocytogenes* and *V*. *cholerae* respectively. *L*. *monocytogenes* forward primer 5’-GCAATTTCGAGGCCTAACCTA and reverse primer 5’–ACTGCGTTGTTAACGTTTGA– 3’ amplifying part of the L-hemolysin gene. *V*. *cholerae* forward primer 5’-ATCGTCAGTTTGGAGCCAGT-3’ and reverse primer 5’-TCGATGCGTTAAACACGAAG-3’for amplification of the hlyA gene using a SYBR green assay (Applied Biosystems). The PCR master mix is made with 5 μL SYBR master mix, 2.8 μL DI water, 0.6 μL 5 μM forward primer, 0.6 μL 5 μM reverse primer, and 1 μL DNA. The run method that is used is 50°C for 2 min, 95°C for 10 min, 95°C 15 seconds followed by 60°C for 1 min for a total of 40 cycles, then 95°C for 15 seconds, 60°C for 1 minute, 95°C for 30 seconds, and finally 60°C for 15 seconds. The DNA standard curve is made with a starting DNA concentration of 1 ng/μL followed by 5 dilutions (3125/1; 652/1; 125/1; 25/1; 5/1).

### Growth analysis of *L*. *monocytogenes*, *S*. *aureus*, *E*. *coli*, and *V*. *cholerae*

*L*. *monocytogenes* was grown on Remel Blood Agar TSA with Sheep Blood (Fisher Scientific R01200) plates and *Staphylococcus aureus* and *Escherichia coli* were grown on Alfa Aesar plain LB agar (Fisher Scientific AAJ61525EQF) plates and incubated in a Fisher Scientific Isotemp incubator at 37°C with 2.3 ± 0.5% CO_2_. Following growth, samples were suspended in DI water and the concentration brought to 10^8^ CFU/mL following McFarland standards. The 10^8^ CFU/mL bacterial suspensions were processed using the methods described above (i.e. bead-beating, protein saver card processing, vortex, or Lyse-It). After processing, 20 μL of lysate was smeared onto the respective bacterial growth plates and incubated over-night. Photographs were taken after 1-night incubation (approximately 12 hours).

For the growth analysis of the four bacteria from 903 protein saver cards, one circle was cut and placed into 500 μL of DI water and incubated at room temperature for 10 minutes. Following incubation, mixing was performed by quickly pipetting up and down to aid in the release of cells from the protein saver card circle. After mixing, one circle in 1 mL DI was sonicated for 10 minutes. Another circle, prepared in the same way, was placed onto the bacterial respective plate to create a “dabbed circle” spot and marked with an ‘X’ and then the circle was placed on the agar for incubation overnight (marked with a circle to indicate where the saver card circle actually was located). Additionally, 20 μL of suspension was aliquoted onto the plate and spread out. Bacterial plates containing the 20 μL aliquot from sonication, room temperature incubation, circle dab, placed circle, and pre, were left in a 37°C incubator overnight and photographs were taken the following morning.

## Results

### The advantages and disadvantages of bead-beating, vortex, 903 protein saver cards, and Lyse-It

To analyze the advantages and disadvantages of the four methods described above, bead-beating, protein saver cards, vortex, and Lyse-It, a table was constructed comparing the various aspects of each respective method (**Table 2**). Cost analysis was determined through purchasing manufactures like Qiagen, Sigma Aldrich, Fisher Scientific, and Lyse-It LLC. For bead-beating 50 stool samples, the cost for the QIAamp DNA Stool Mini Kit is $277. This allows for total stool DNA isolation from both liquid or solid samples. Following the procedure described for bead-beating, the number of steps was 23 totaling many hours for preparing just 16–24 samples. The benefit of this method was that the pathogenic bacteria was dead, however, only the DNA was viable for future analysis like PCR, as other intracellular components and cellular debris were discarded throughout the procedure. In comparison, a vortex, which is relatively inexpensive for a long-lasting piece of equipment, can be used for a variety of sample types like solid and liquid stools and other laboratory applications. The two main disadvantages to using a vortex on pathogenic bacteria is that the bacteria does not 100% die from the vigorous mixing and thus, if samples are needed to be shipped through the mail or via airplane, a cold chain and proper shipping and handling procedures must be followed. Unlike bead-beating and vortex, 903 protein saver cards and Lyse-It are rapid and do not require a cold chain or special shipping requirements to transport bacterial processed samples. Lyse-It comes with a starter kit bundle for $725. Additional options for slides and other consumables can be found at www.Lyse-It.com. The starter kit includes everything that is needed for a rapid 30 second– 60 second lysing. Additional sample chambers (isolators) and lysing slides can be purchased in varying quantities at a slight additional cost. For the protein saver cards, 100 cards can be purchased for $173, totaling some 500 samples to be safely shipped with subsequent DNA detection and amplification through qPCR.

**Table 2:** The four methods studied.

Method	Bead-Beating	Protein Saver Cards	Vortex	Lyse-It
**Cost**	50 samples—$277	100 cards– $173	1 Vortex—$250-$500	100 slides– $875
**# of Steps**	23	6	6	6
**Time**	Hours	30 minutes	Minutes	Minutes
**Shelf-Life Product**	Years	Years	Years	Years
**Product Cold Chain Required**	No	No	No	No
**# of Equipment Pieces**	14	3	1	4
**Ease of Use**	Complex	Medium	Very Easy	Very Easy
**Lysate Pathogenicity**	N/A	Dead	Alive	Dead
**Lysing Ability to Fragment DNA**	Yes	No	No	Yes
**Bacteria Shipment Cold Chain Required**	N/A	No	Yes	No

### Confirmation of gram-positive and–negative growth inactivity using Lyse-It, 60°C conventional heating, and 903 protein saver cards

To investigate bacterial growth of Gram-positive and–negative bacteria, various conditions using Lyse-It, vortex, sonication, or 60°C conventional heating times were tested. Lyse-It microwave powers of 30% and 50%, 270 and 450W respectively, with irradiation times of 30 and 60 seconds were tested. For the other three methods, 1 minute, 4 minutes, 8 minutes, and 15 minutes were chosen as 1 minute was comparable to the 60 seconds microwave irradiation time and 8 minutes of the conventional heating time used in the standard protocol for rehydration and extraction of sample from the protein saver cards. The growth for *L*. *monocytogenes*, *S*. *aureus*, and *E*. *coli* can be seen in **Fig 1** and **S2 Fig** for *V*. *cholerae*. In all cases, the *pre (time (t) = 0)* was plated from the 10^8^ CFU/mL bacterial suspensions. For Lyse-It, it was found that for the Gram-positive bacteria *L*. *monocytogenes* and *S*. *aureus* both the microwave irradiation power and time needed to be greater than 30%, 60 seconds (**Fig 1 Top panel and bottom left panel**) for complete kill of the bacteria. For both *L*. *monocytogenes* and *S*. *aureus*, 30% and 30 seconds only decreased the quantity of viable organisms as shown by the dark streaking showing through on the blood plates and bacterial growth on the LB plates for *L*. *monocytogenes* and *S*. *aureus* respectively. On the contrary, for *E*. *coli* and *V*. *cholerae*, a Gram-negative organism, 30% power 30 seconds was enough to completely kill the bacteria’s ability to grow (**Fig 1 bottom right panel**). Conversely for *L*. *monocytogenes*, *E*. *coli*, and *S*. *aureus* the four tested times for sonication and vortex *did not* inactivate any of the organisms’ ability to grow as shown by the dark coloration on the blood plates and growth on the LB plates.

**Fig 2:**
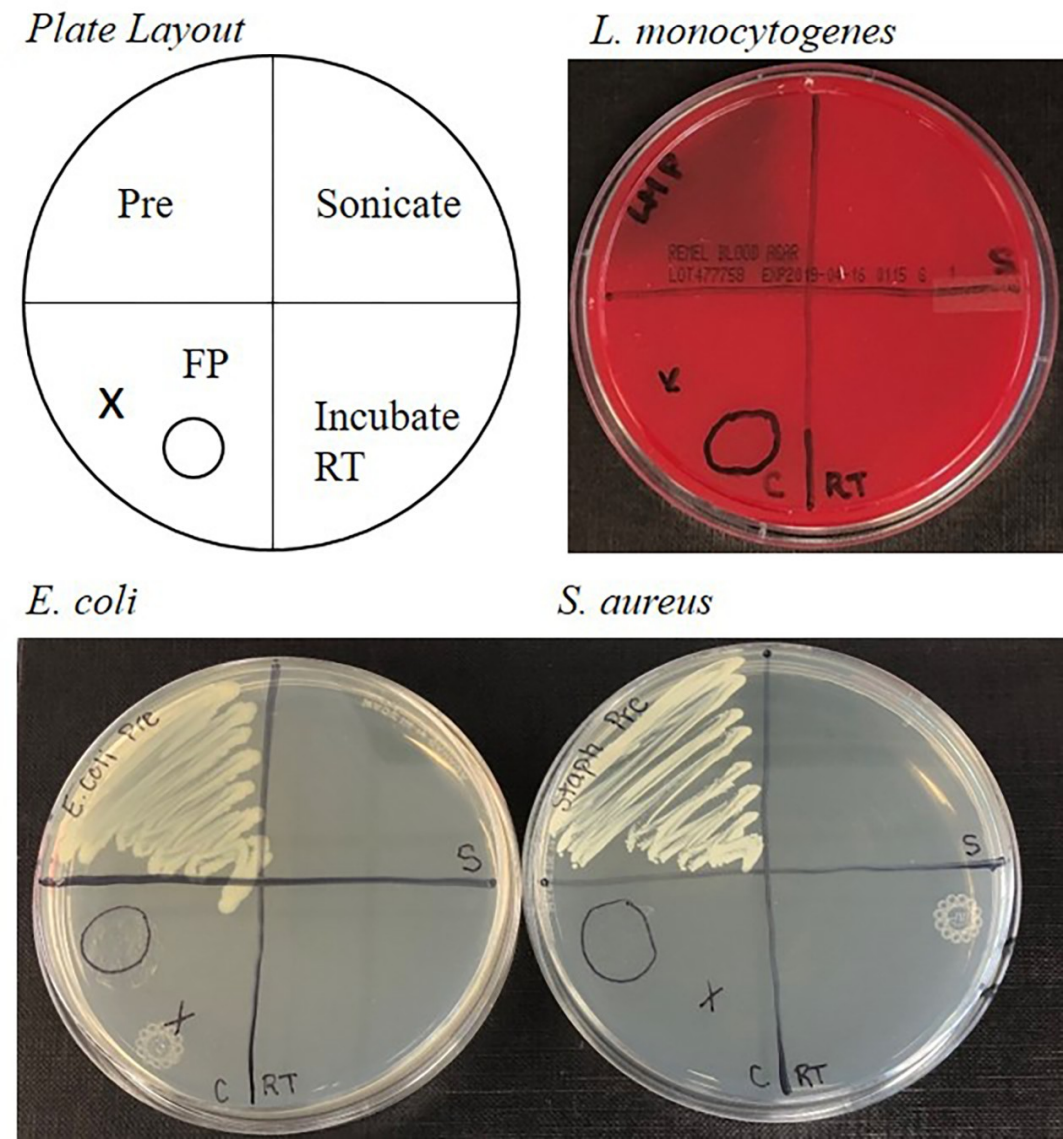
Growth of *L*. *monocytogenes*, *E*. *coli*, and *S*. *aureus* bacteria following extraction from 903 protein saver cards. Pre = 10^8^ CFU/mL suspension without further processing. FP (or C) = 903 Protein Saver Card Circles, RT = Room temperature incubation, S = sonication post room temperature incubation.

To demonstrate that live pathogenic bacteria is not viable after drying on protein saver cards, aliquots of *L*. *monocytogenes*, *S*. *aureus*, *E*. *coli*, and *V*. *cholerae* were placed onto designated circles of the cards and allowed to dry for one month. This drying time was chosen to demonstrate that 1) the bacteria was completely dry and safe for shipping and 2) post extraction of cells from the protein saver cards, the DNA was still viable for qPCR. As shown in **Fig 2**, after rehydration through a 10-minute incubation period and/or sonication with aliquots of rehydration suspension, no bacterial growth was seen as compared to healthy bacteria for *L*. *monocytogenes*, *S*. *aureus*, *E*. *coli* or *S*. *aureus*. Similar results were seen with *V*. *cholerae* in **S3 Fig**.

### DNA from *V*. *cholerae* and *L*. *monocytogenes* is viable for qPCR following drying on 903 protein saver cards

**Fig 3:**
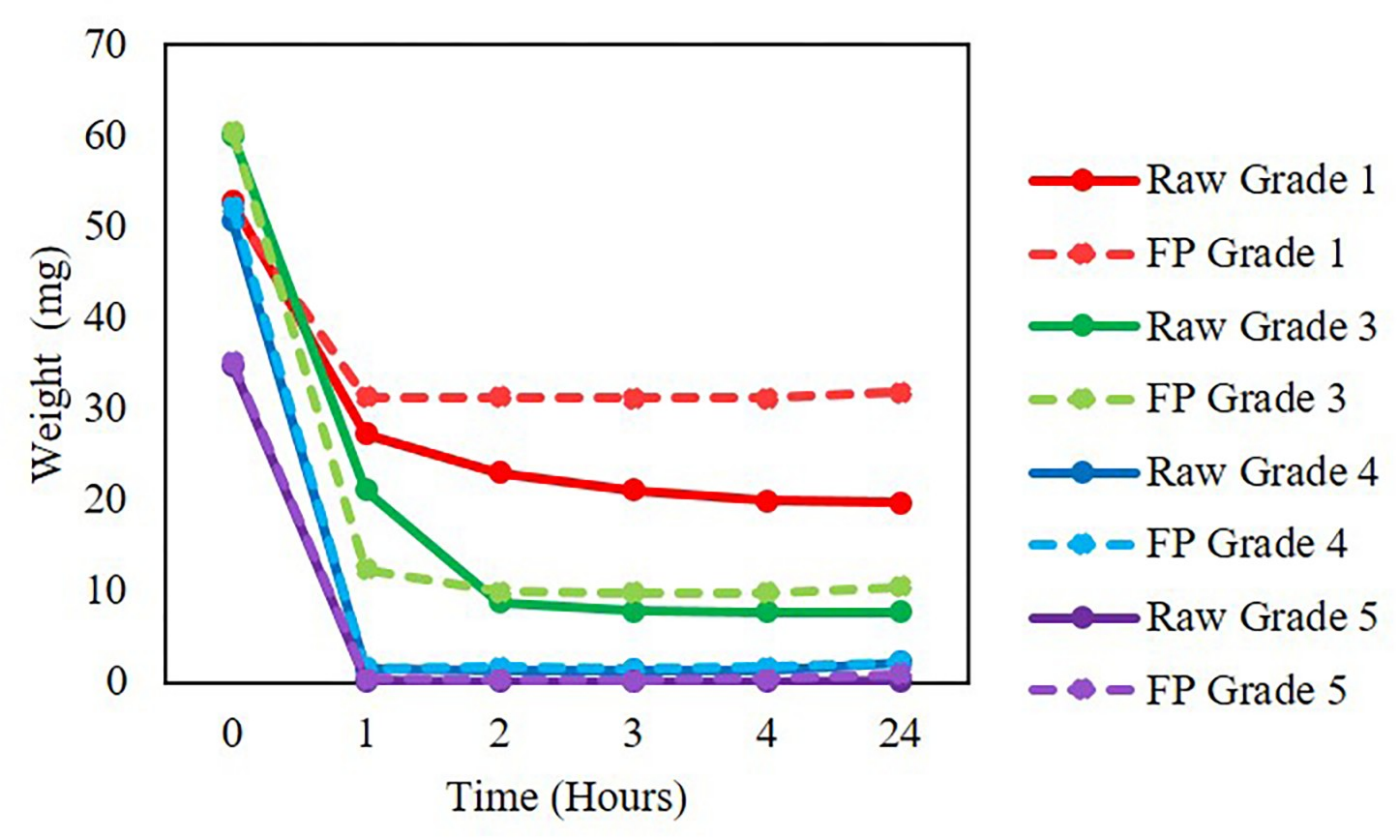
Raw *V*. *cholerae* in stool and *V*. *cholerae* in stool aliquoted on to 903 protein saver cards drying times. FP = 903 Protein Saver Card Circles.

**Fig 4:**
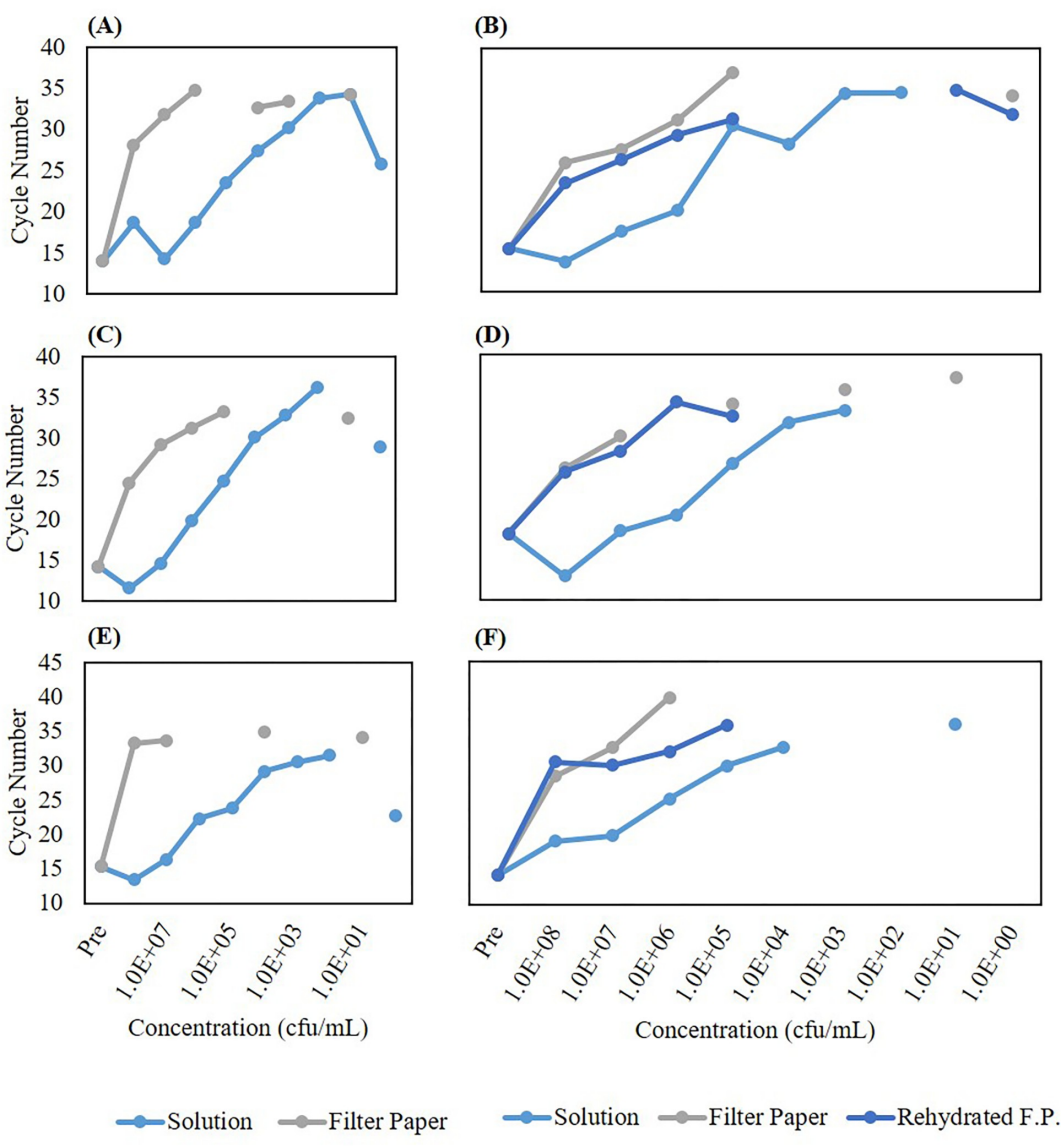
qPCR cycle number for various concentrations of *V*. *cholerae* (Left) and *L*. *monocytogenes* (Right) in solution as compared to extracted off of 903 filter paper post 5 minutes vortex **(A, B)**, 5 minutes sonication **(C, D)**, or microwave irradiation **(E, F)**. **(A, C, E)** A lower cycle number is obtained when *V*. *cholerae* is in solution as compared to releasing cells from filter using vortex, sonication, or 30% power, 60 second microwave irradiation. **(B, D, F)** When the filter is first rehydrated in water and then vortexed, sonicated, or microwaved at 50% power, 60 seconds, more extraction of *L*. *monocytogenes* is detected through qPCR. FP (Filter Paper) = 903 Protein Saver Card.

We tested three ways to extract *V*. *cholerae* and *L*. *monocytogenes* bacterial cells from the protein saver cards–vortex, sonication, or Lyse-It with and without pre-hydration. After the different lysing methods and thawing from storage, samples at varying concentration (10^8^–10^0^ CFU/mL) were run on qPCR and compared against bacterial suspensions of the same concentration. The weights for the stool samples both raw and on the protein saver cards were measured post sample drying. Weights were compared to those of initial weights and it was found that post drying, the weight decreased significantly. Therefore, a preliminary sample drying experiment was performed where weights ranging from 1–24 hours on the filter paper were calculated. It was found that after 1 hour, sample weights did not vary significantly (**Fig 3**) i.e. the solvent had evaporated. The solution cycle number was the baseline to see how well the other cellular extraction processes worked. In all cases, the cycle number for the solution was significantly lower than that for vortex, sonication, and microwave irradiation for both *V*. *cholerae* and *L*. *monocytogenes* (**Fig 4**). We attribute the high cycle number from the protein saver cards even after the trial of cells being rehydrated to the embedding and sticking of the cells to the protein saver cards and not being efficiently released into the water. It was seen that *L*. *monocytogenes* cells that were pre-hydrated and then lysed through the various methods did lower the cycle number. Additionally, at lower concentrations (10^5^ CFU and below) for all samples, there was no cycle number recorded. We attributed no cycle number recording to the sensitivity of the qPCR instrument. Thus, *we confirmed* that even-though bacteria are dried and dead (cannot regrow) on the 903 saver cards, the DNA was still viable for detection via qPCR with the three different cellular extraction methods.

### Centrifuge filters are required to remove qPCR contaminants, whole bacterial cells, and see the difference between standard microwave irradiation and using Lyse-It

**Fig 5:**
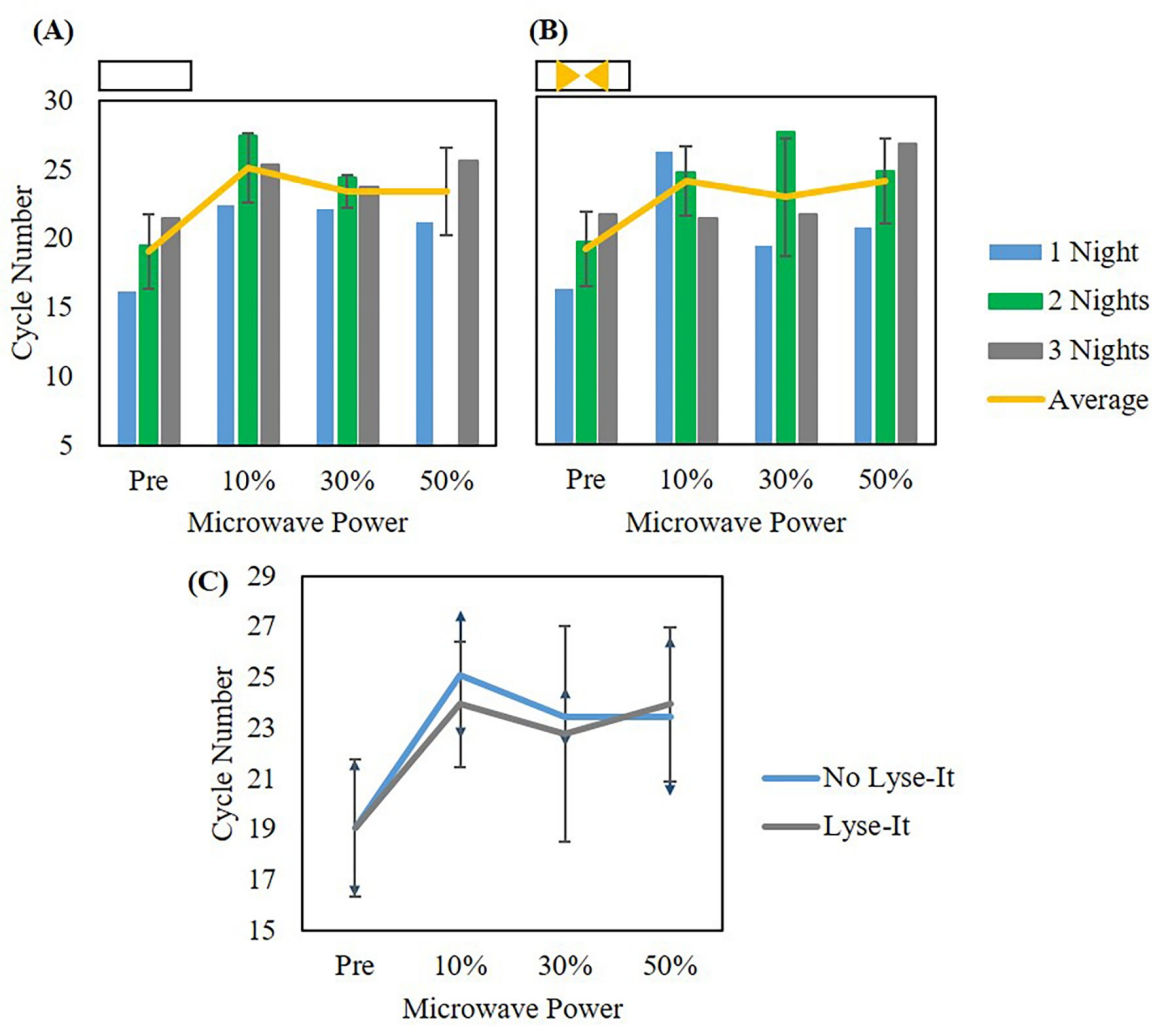
qPCR of *L*. *monocytogenes* detected from ground spinach with and without Lyse-It and no filtration. **(A)** Cycle number comparisons of three incubation times of extraction of *L*. *monocytogenes* without Lyse-It. **(B)** Cycle number comparisons of three incubation times with extraction of *L*. *monocytogenes* by Lyse-It. In both **(A)** and **(B)**, there is no statistical difference in cycle number as incubation time increases. Therefore, cycle numbers were averaged across all incubation times to create **(C)**. In terms of *L*. *monocytogenes* extraction and detection through qPCR, there is no statistical difference in cycle number with or without the use of gold Lyse-It. Note: No Lyse-It means simply heating in a microwave cavity.

In addition to testing cellular extraction methods for cellular release from 903 protein saver cards, we tested how effective Lyse-It was for cellular extraction as compared to the use of standard microwave lysing (i.e. as compared to simply heating in a microwave). This experiment was performed where *L*. *monocytogenes* was grown and then suspended in DI water to 10^8^ CFU/mL. The suspension was left in a 37°C incubator for 24, 48, or 72 hours, 1–3 nights respectively. After each time point, an aliquot of the suspension was pipetted onto individual circles of the protein saver cards and allowed to dry at room temperature for over 1 month. Previously, it was shown that extracellular DNA was visible after making bacterial suspensions.[16] However, it was only until after days to a week that *L*. *monocytogenes* cells osmotically lyse and the genomic DNA began to degrade.[16] Following one month, the saver cards containing the sample were rehydrated with 1.25 mL DI water and mixed to aid in the release of bacterial cells. The mixed suspension was microwave irradiated with or without Lyse-It at 10%, 30%, or 50% power for 60 seconds. The lysate was removed from the slide, cooled at room temperature, and stored at -20°C until qPCR was performed. Prior to qPCR, there were no additional steps taken to purify the DNA from the sample. Cycle numbers were graphed with respect to incubation time (1–3 nights) and microwave power with (**Fig 5B**) and without Lyse-It (**Fig 5A**). It was seen that incubation time did not affect the detection and subsequent amplification of the *L*. *monocytogenes* L- hemolysin gene with or without Lyse-It. Additionally, when comparing microwave power between no Lyse-It and Lyse-It, there appeared to be no statistical difference between the two techniques (**Fig 5C**).

**Fig 6:**
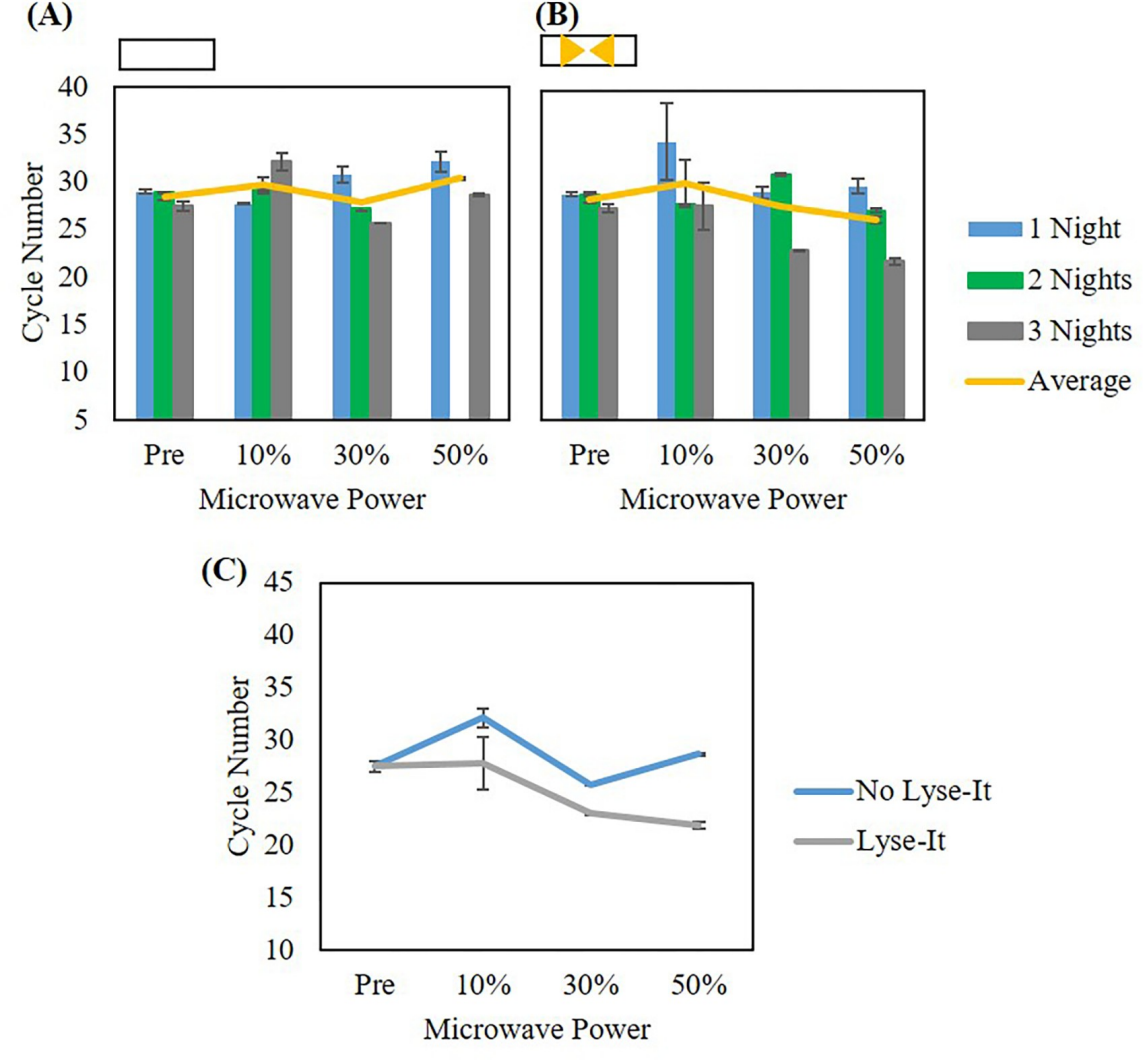
qPCR of *L*. *monocytogenes* detected from ground spinach with and without Lyse-It after filtering the product post lysing. **(A)** Cycle number comparisons of three incubation times of extraction of *L*. *monocytogenes* without Lyse-It. **(B)** Cycle number comparisons of three incubation times with extraction of *L*. *monocytogenes* by Lyse-It. **(C)** Average of cycle numbers for the three incubation times. Statistical differences are seen in both the microwave power used for *L*. *monocytogenes* extraction as well as for no Lyse-It versus Lyse-It. Note: No Lyse-It means simply heating in a microwave cavity.

This led us to investigate the use of 0.45 μm pore centrifuge filters to separate out extracted and fragmented DNA from the two techniques. By not centrifuge filtering the lysate samples, intact cells that may have not been lysed through microwave irradiation were thought to be lysed in the PCR machine during the denaturation step (approximately 95°C). Thus, the same experiment was performed with the use of centrifuge filters post standard microwave irradiation and with using Lyse-It. Here, it was seen that incubation time did not matter for qPCR detection and subsequent DNA amplification using either standard microwave irradiation or Lyse-It (**Fig 6A and 6B**). However, when looking at the difference between standard microwave irradiation as compared to Lyse-It, Lyse-It was able to *significantly release more cells and extract more DNA* for qPCR shown by the lower cycle number (**Fig 6C**). Additionally, as microwave power increased, more cells were released, and subsequently more lysed cells were available for qPCR DNA detection (**Figs 5C and 6C**).

### Bead-beating, vortex, sonication, and Lyse-It are all capable for the extraction of various stool grade *V*. *cholerae* from raw samples and 903 protein saver cards

**Fig 7:**
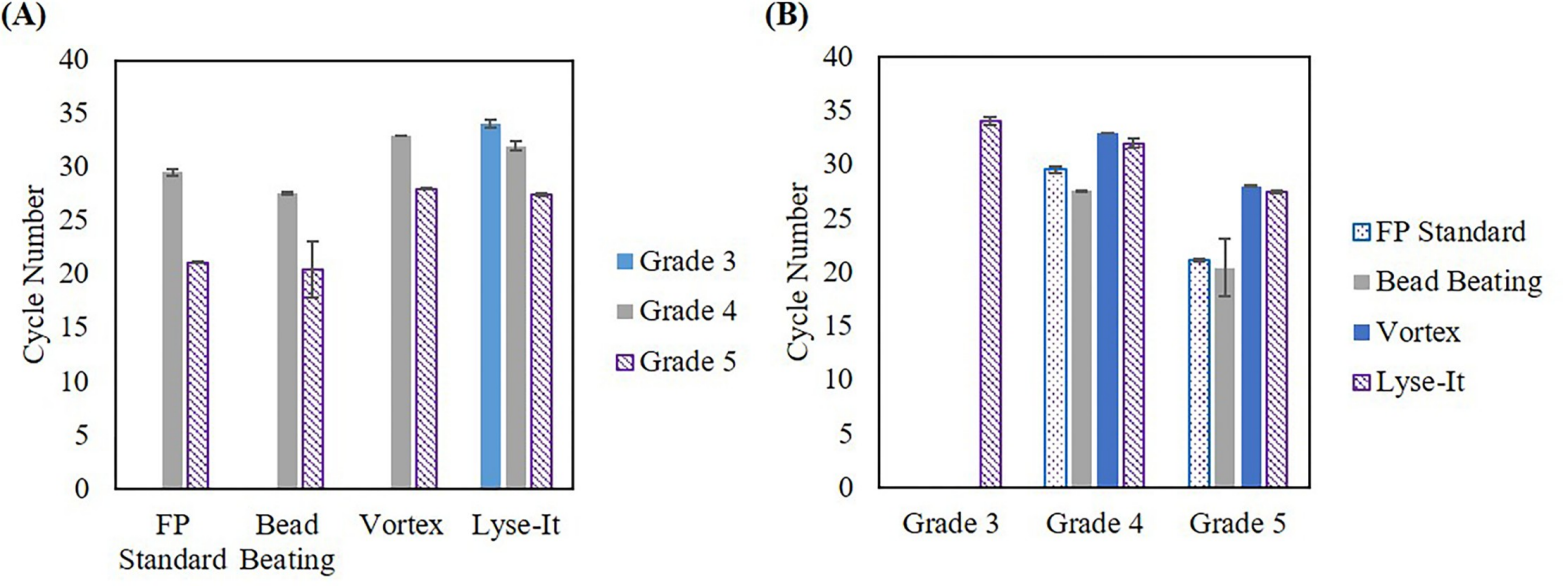
qPCR results of *V*. *cholerae* extracted from raw stool samples using various processing techniques. **(A)** Processing comparison **(B)** Stool grade comparison. Lyse-It was seen to be more effective at extracting DNA versus the other techniques studied.

**Fig 8:**
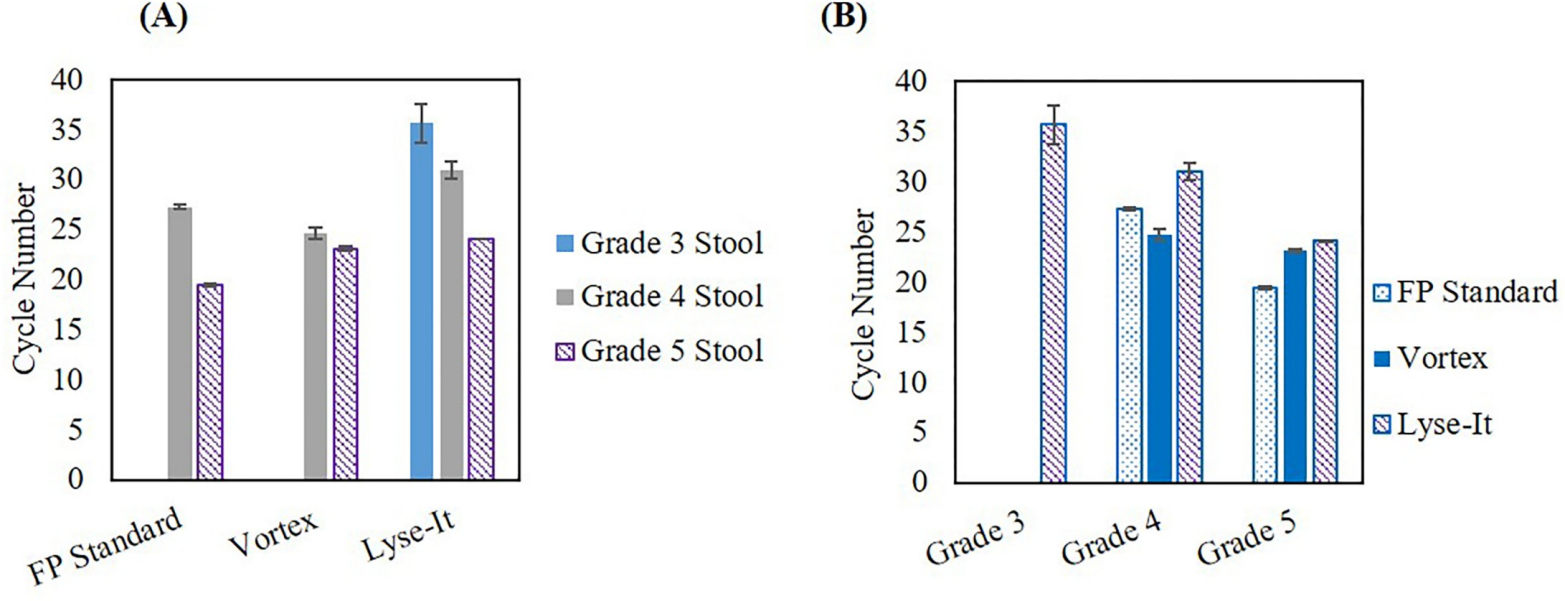
qPCR results of *V*. *cholerae* extracted from 903 protein saver cards using various processing techniques. **(A)** Processing comparison **(B)** Stool grade comparison. Lyse-It was seen to be more effective at extracting DNA versus the other techniques.

Utilizing the data obtained from using the 0.45 μm centrifuge filters, we looked at both DNA extraction and detection using four methods, namely–the 903 protein saver card method, bead-beating, vortex, and Lyse-It (30% power, 60 seconds run time). It was determined that all methods were able to achieve DNA extraction and detection from grades 4 and 5 stools through qPCR (**Fig 7**). To be brief, *V*. *cholerae* infections severity can be seen/determined through fecal analysis. There are five stool grades (Grades 1–5) that are associated with *V*. *cholerae* infections. Grade 5 stools are the most severe grade as these samples contain the highest amount of *V*. *cholerae* and are visually considered “rice water stools”. As the stool grade numerically decreases, the overall concentration of *V*. *cholerae* decreases and the fecal sample visually appears to be a healthier and progressively solid sample. Therefore, as stool grade increases and thus the *V*. *cholerae* concentration increases, a smaller qPCR cycle number is expected. In most cases, this was confirmed, subsequently demonstrating that these techniques are capable of bacterial cell and DNA extraction from a wide range of bacterial concentrations. Interestingly, the Lyse-It technique was able to lyse more *V*. *cholerae* cells in grade 3 stools and thus genomic DNA was able to be detected and amplified through qPCR, as compared to the other three methods studied, which did not yield viable DNA from the grade 3 stool. Additionally, similar results were achieved when extracting and detecting *V*. *cholerae* from the protein saver cards (**Fig 8**), where more DNA was recovered using the Lyse-It technology. These results clearly demonstrate that even-though the bacteria in previously frozen stool or on protein saver cards in dead, the genomic DNA is still viable and able to be recovered through the four techniques described above, albeit, much more efficiently with the Lyse-It technology.

## Discussion

In settings where technology, doctors, transportation to a medical facility, instrumentation, and cost is limited, it is imperative that pathogenic samples can be analyzed in a safe and timely fashion. For samples where there is known or suspected bacterial or viral pathogenicity to humans or animals, there are strict shipping guidelines to adhere to. Herein, we have demonstrated two safe, inexpensive, low technology, low complexity, fast and efficient methods namely the– 903 protein saver cards and Lyse-It—for neutralizing and rendering non-infectious Gram-positive and -negative pathogenic bacteria for safe shipping and handling, as well as a further two methods for the extraction of bacterial cells and DNA for subsequent qPCR detection.

We have compared four methods in terms of equipment cost, shelf-life of the product, cold chain requirements, and the ability to fragment DNA amongst others. Additionally, we have been able to demonstrate that lowering the amount of sample needed for *V*. *cholerae* detection can be reduced to milligram quantities instead of at least 1 gram of sample currently being used for colony counting.

One of the most important aspects of this research was the ability of 60°C conventional heating, the Lyse-It technology, and 903 protein saver cards to inactivate *L*. *monocytogenes*, *S*. *aureus*, and *E*. *coli* growth in their select media. This is incredibly beneficial as the bacteria have been essentially neutralized for safe shipping and handling across the United States and internationally. Additionally, the samples on protein saver cards preserve bacterial genomic DNA for future qPCR detection. Lyse-It, as well as the 903 protein saver cards, can be used in third world countries as both technologies do not require a cold chain and can render the growth of bacteria inactive. Lyse-It also has the capability to extract and lyse cells from a variety of medias, including the protein saver cards, while subsequently fragmenting DNA and proteins for downstream detection methods.

Finally, these methods can serve many purposes like the neutralization of pathogenic organisms, intracellular component extraction, and preservation of intracellular components like genomic DNA for future detection. Further, while beyond the scope of this manuscript, our studies of the Lyse-It technology demonstrates that it can substantially lower and inactivate DNA/RNA nuclease activity, which we feel is ideal for long-term sample stability. Further details will be reported in due course.

## Supporting information

S1 Fig 903 protein saver card cut out grade stools versus raw stool samples. (TIF)

S2 Fig *V. cholerae* growth after Lyse-It 30% and 50% power for 30 and 60 seconds and 1 minutes of 60°C conventional heating. (TIF)

S3 Fig *V. cholerae* growth after drying on 903 protein saver cards. (TIF)

S1 Schematic Extraction of *V. cholerae* from raw and 903 protein saver cares using standard 903 processing, bead beating, vortex, and using Lyse-It. (TIF)
