# Supplementary figures and images for: A comparison of Lyse-It to other cellular sample preparation, bacterial lysing, and DNA fragmentation technologies
Source: PLoS One. 2019 Jul 23;14(7):e0220102. doi: 10.1371/journal.pone.0220102 (PMC6650070; doi:10.1371/journal.pone.0220102)

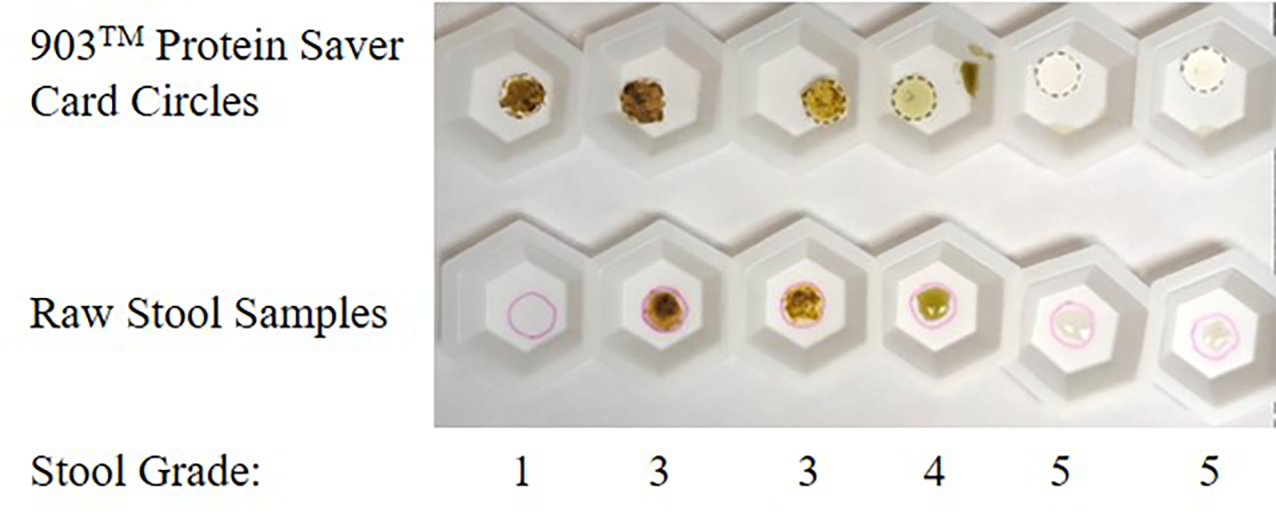

Supplement: S1 Fig — (TIF) [file pone.0220102.s001.tif]

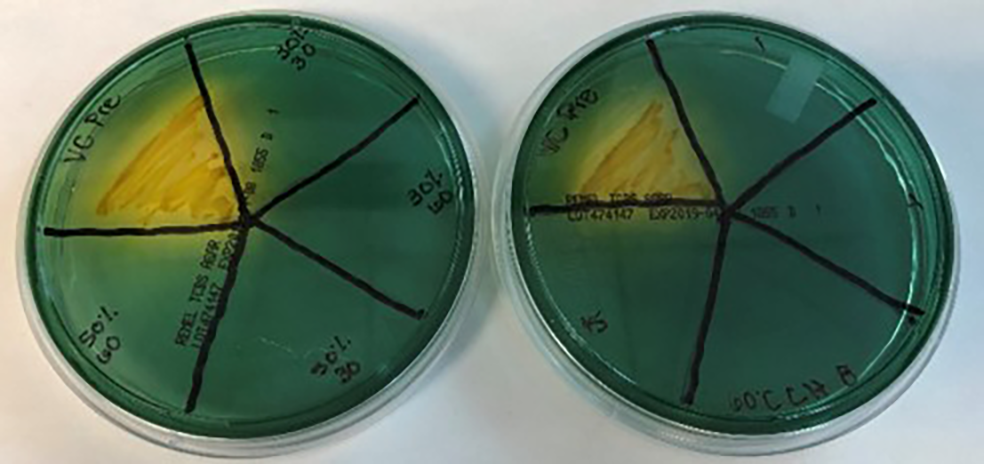

Supplement: S2 Fig — (TIF) [file pone.0220102.s002.tif]

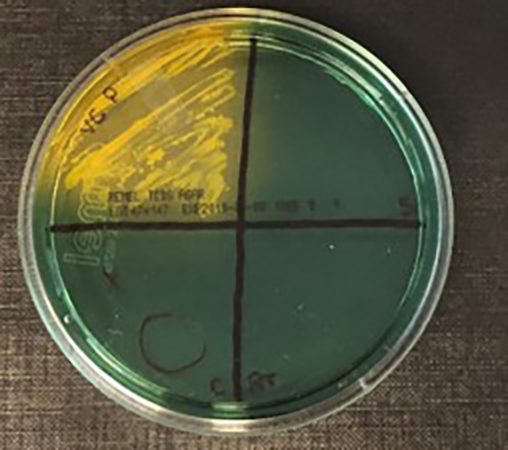

Supplement: S3 Fig — (TIF) [file pone.0220102.s003.tif]

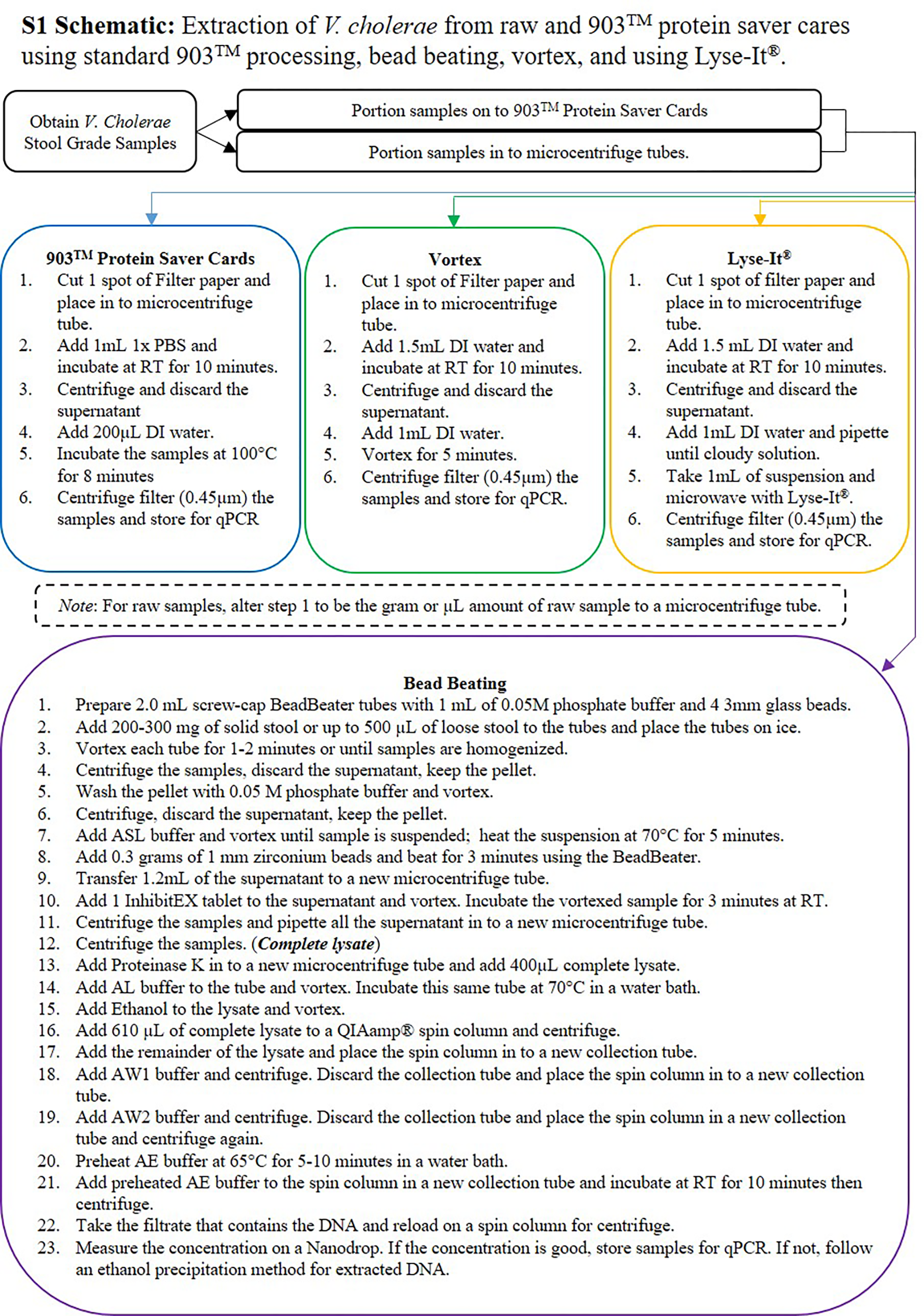

Supplement: S1 Schematic — (TIF) [file pone.0220102.s004.tif]
